# The Dual Role of the Glycolipid Envelope in Different Cell Types of the Multicellular Cyanobacterium *Anabaena variabilis* ATCC 29413

**DOI:** 10.3389/fmicb.2021.645028

**Published:** 2021-04-09

**Authors:** Ritu Garg, Iris Maldener

**Affiliations:** Institute of Microbiology and Infection Medicine, Organismic Interactions, University of Tübingen, Tübingen, Germany

**Keywords:** cyanobacteria, *Anabaena*, heterocyst, akinete, HglB, glycolipids, stress tolerance, evolution

## Abstract

*Anabaena variabilis* is a filamentous cyanobacterium that is capable to differentiate specialized cells, the heterocysts and akinetes, to survive under different stress conditions. Under nitrogen limited condition, heterocysts provide the filament with nitrogen by fixing N_2_. Akinetes are spore-like dormant cells that allow survival during adverse environmental conditions. Both cell types are characterized by the presence of a thick multilayered envelope, including a glycolipid layer. While in the heterocyst this glycolipid layer is required for the maintenance of a microoxic environment and nitrogen fixation, its function in akinetes is completely unknown. Therefore, we constructed a mutant deficient in glycolipid synthesis and investigated the performance of heterocysts and akinetes in that mutant strain. We chose to delete the gene *Ava_2595*, which is homolog to the known *hglB* gene, encoding a putative polyketide synthase previously shown to be involved in heterocyst glycolipid synthesis in *Anabaena* sp. PCC 7120, a species which does not form akinetes. Under the respective conditions, the *Ava_2595* null mutant strain formed aberrant heterocysts and akinete-like cells, in which the specific glycolipid layers were absent. This confirmed firstly that both cell types use a glycolipid of identical chemical composition in their special envelopes and, secondly, that HglB is essential for glycolipid synthesis in both types of differentiated cells. As a consequence, the mutant was not able to fix N_2_ and to grow under diazotrophic conditions. Furthermore, the akinetes lacking the glycolipids showed a severely reduced tolerance to stress conditions, but could germinate normally under standard conditions. This demonstrates the importance of the glycolipid layer for the ability of akinetes as spore-like dormant cells to withstand freezing, desiccation, oxidative stress and attack by lytic enzymes. Our study established the dual role of the glycolipid layer in fulfilling different functions in the evolutionary-related specialized cells of cyanobacteria. It also indicates the existence of a common pathway involving HglB for the synthesis of glycolipids in heterocysts and akinetes.

## Introduction

To cope with various stress conditions, cyanobacteria require adaptation and survival strategies. Cell differentiation is key for successful survival in harsh and changing environmental conditions (Rippka and Herdman, 1985; Flores and Herrero, 2010). The planktonic freshwater filamentous cyanobacterium *Anabaena variabilis* ATCC 29413 can undergo a variety of cellular differentiation processes forming motile hormogonia, nitrogen-fixing heterocysts, and spore-like dormant cells called akinetes, and therefore, serves as a model organism to study the cell differentiation process in prokaryotes (Flores and Herrero, 2010; Maldener et al., 2014). The related species *Anabaena* sp. PCC 7120, which does not form akinetes, is the model organism to study heterocyst differentiation.

In *A. variabilis*, the nitrogen (N_2_) fixing heterocysts appear in a semi-regular pattern along the filament in response to insufficient supply of combined nitrogen under aerobic conditions (Fay, 1992; Muro-Pastor and Maldener, 2019). Nitrogen fixation is mediated by the enzyme nitrogenase, which is highly sensitive to oxygen. To protect the nitrogenase, a microoxic environment is maintained in the heterocysts by inactivation of oxygen-evolving photosynthesis, by increased respiration, and by formation of an additional multilayered cell envelope outside the cell wall (Murry et al., 1984; Walsby, 1985; Murry and Wolk, 1989; Valladares et al., 2003, 2007; Magnuson, 2019). The heterocyst envelope is composed of two layers: an inner heterocyst-specific glycolipid (HGL) layer, which acts as a barrier to oxygen and limits its diffusion into the heterocyst, and an outermost heterocyst envelope polysaccharide (HEP) layer, which protects the HGL layer from physical damage (Wolk et al., 1994; Adams, 2000; Maldener et al., 2014; Muro-Pastor and Maldener, 2019).

Akinetes are spore-like non-motile cells that differentiate from the vegetative cells in response to diverse environmental factors including changes in light intensity, temperature, and nutrient deficiency. Akinetes can endure dryness and cold, while being vulnerable to high temperatures unlike spores, which are heat resistant. For this reason, they are just referred to as “spore-like” cells. Akinetes differ from the vegetative cells by their cellular structure, composition, and morphology. Akinetes ensure a longer period of survival to cyanobacteria under harsh and unfavorable conditions due to their resistance to cold and desiccation, and serve a perennation role (Yamamoto, 1976; Kaplan-Levy et al., 2010). Akinete formation is a transient process. When environmental conditions are favorable for growth, akinetes germinate into vegetative cells and start their life cycle over (Maldener et al., 2014). The light, temperature, and nutrient conditions favorable for growth appear to be the major stimulus for akinete germination (Yamamoto, 1976; Van Dok and Hart, 1997; Perez et al., 2016; Sukenik et al., 2018). Compared to akinete differentiation, which takes several days up to weeks, germination was observed to occur much faster in just few hours (Perez et al., 2018).

The process of akinete differentiation is characterized by the transient accumulation of storage compounds (such as glycogen, cyanophycin, lipids and nucleic acids), reduction of metabolic activities and formation of a thick multilayered envelope (Sutherland et al., 1979; Sukenik et al., 2012; Perez et al., 2016, 2018). This specialized envelope is composed of an outermost polysaccharide layer and inner layers of glycolipids, similar in composition to that of the heterocyst envelope (Cardemil and Wolk, 1976, 1981; Reddy, 1983; Soriente et al., 1993; Wolk et al., 1994; Wolk, 1996; Perez et al., 2018; Qiu et al., 2019). Whilst the function of the HGL layer is well understood in heterocysts (Haury and Wolk, 1978; Ernst et al., 1992; Fay, 1992), its role in akinetes and stress survival is unknown. Furthermore, the akinete envelope has some extra laminated layers, the function of which remains unknown (Braune, 1980; Nichols and Adams, 1982; Perez et al., 2018). Due to high similarity between their cell envelopes, akinetes have been supposed to be the evolutionary precursors of heterocysts (Wolk et al., 1994).

Several genes encoding the enzymes responsible for HEP and HGL synthesis in the heterocyst envelope have been identified [summarized in Nicolaisen et al. (2009); Maldener et al. (2014)]. The clustered genes *alr5351–alr5357* (also known as *hglE_*A*_, hglD, hglC*, and *hglB)* in *Anabaena* sp. PCC 7120 (Black and Wolk, 1994; Bauer et al., 1997; Awai et al., 2009; Saito and Awai, 2020) and the *hglE* gene in *Nostoc punctiforme* (Campbell et al., 1997) are associated with glycolipid layer formation. The *hglBCDE*_*A*_ gene cluster encodes putative enzymes for the biosynthesis of HGL aglycone (Fan et al., 2005; Awai et al., 2009; Saito and Awai, 2020). The *devBCA* gene cluster has been shown to be required for HGL export in *Anabaena* sp. PCC 7120 and *A. variabilis* (Fiedler et al., 1998a, b; Staron et al., 2011). Similarly, the gene cluster *hgdABC* is essential for proper HGL layer deposition in the heterocyst envelope in *Anabaena* sp. PCC 7120 (Fan et al., 2005; Shvarev et al., 2018).

Several heterocyst genes have also been found associated with akinete formation. For instance, the *hepA* gene, encoding a putative polysaccharide exporter, is essential for correct heterocyst envelope formation and consequently, the *A. variabilis hepA*-mutant forms an abnormal akinete envelope (Leganés, 1994). The overexpression of the heterocyst regulatory gene *devR* results in enhanced akinete differentiation in *N. punctiforme* (Campbell et al., 1996). In *Nostoc ellipsosporum*, deletion of the regulatory gene *hetR* inhibited both heterocyst and akinete differentiation (Leganés et al., 1994). However, a *hetR* mutant of *N. punctiforme* under phosphate starvation conditions could form large akinete-like cold-resistant cells (Wong and Meeks, 2002). These studies indicate a role for heterocyst genes in akinete formation, suggesting a common pathway regulating their differentiation.

The *hglB* gene [also known as *hetM* (Black and Wolk, 1994) or *alr5357*] encodes a putative polyketide synthase required for the synthesis of the glycolipid aglycones. HglB possesses two functional domains, an N-terminal acyl carrier protein (ACP) domain and a C-terminal thioester reductase (TER) domain (Black and Wolk, 1994; Bauer et al., 1997; Awai et al., 2009). The characterization of a *hglB* mutant of *Anabaena* sp. PCC 7120 (Maldener et al., 2003) demonstrated the role of this gene in HGL synthesis during heterocyst differentiation. *A. variabilis* harbors a gene in its genome with high similarity to *hglB*. To investigate the role of this *hglB* homolog in the synthesis of the glycolipid layer of akinetes, we performed a mutational analysis in *A. variabilis.* As expected, the HGL layer of heterocysts is absent in the *hglB* mutant of *A. variabilis*. But also, distinct laminated layers are absent in the mutant akinetes. These aberrant akinetes lose their resistance against various stress conditions implying a different function of the glycolipid layer in heterocysts and akinetes. With this study, we were also able to show that envelope formation requires the same biosynthetic pathway in akinetes and heterocysts indicating an evolutionary relationship between both differentiation processes.

## Materials and Methods

### Bacterial Strains and Culture Conditions

Vegetative cultures of *Anabaena variabilis* ATCC 29413 strain FD (Currier and Wolk, 1979; Thiel et al., 2014) and derived mutant strains (Supplementary Table 1) were grown photoautotrophically under continuous illumination (17–22 μmol photons m^–2^ s^–1^) at 28°C with shaking at 120 rpm in standard medium of Allen and Arnon (1955) diluted 4-fold with water (AA/4) and supplemented with 5 mM KNO_3_. The solid media remained undiluted with 1.5% (w/v) Difco Agar. For growing the mutant strain, 50 μg ml^–1^ neomycin was added to the medium. To induce heterocyst differentiation, the exponentially growing cultures (OD_750 *nm*_ 0.4–0.5) were harvested and washed three times in nitrate-free AA/4 medium, resuspended in same medium equal to the original volume, and cultivated under nitrogen depleted conditions.

*Escherichia coli* strains were grown in lysogeny broth (LB) medium at 37°C, supplemented with the following antibiotics: 50 μg ml^–1^ kanamycin (Km), 25 μg ml^–1^ chloramphenicol (Cm), 25 μg ml^–1^ streptomycin (Sm) and 100 μg ml^–1^ spectinomycin (Sp), when required. For growth on solid medium, 1.5% (w/v) agar was added. The *E. coli* strain Top 10 was used as a host for plasmid constructions. For triparental conjugation, the *E. coli* strain J53 (bearing the conjugative plasmid RP4), strain HB101 (bearing the helper plasmid pRL528 and the cargo plasmid), and the wild type (WT) *A. variabilis* culture were used (Maldener et al., 1991; Supplementary Table 1).

### Mutant Construction

To construct the *hglB* mutant in *A. variabilis*, the gene *Ava_2595* (*hglB*) was inactivated by insertion of the neomycin-resistance-conferring cassette (C.K3) into the genome by double-crossover homologous recombination (Elhai and Wolk, 1988). For this, the left- and right-flanking regions of 500 bp from *hglB* were amplified in PCR using primers 1979 and 1980, and 1983 and 1984 (see Supplementary Table 2 for primers) using genomic DNA as template and high fidelity Q5-polymerase (NEB, Ipswich, MA, United States). The C.K3 cassette was amplified from the plasmid pIM74 (Fiedler et al., 1998b) using primers 1981 and 1982. All PCR products were fused into the PstI digested suicide vector pRL271 (Supplementary Table 1) using Gibson assembly (Gibson et al., 2009). The resulting plasmid pIM752 was transferred into WT *A. variabilis* cells via triparental mating followed by the selection of clones on neomycin and 5% sucrose-containing agar plates (Maldener et al., 1991). Several clones were checked for full segregation of the mutated gene by colony PCR using primers 2534 and 2535. One of these genotypically verified and identical mutant clones was chosen for our study and named as DR752.

### Akinete Differentiation and Germination

For akinete induction, BG11 medium containing NaNO_3_ (Rippka et al., 1979) was used, as the differentiation of akinetes was better observed in this medium compared to AA/4. The late-exponentially grown cultures (OD_750nm_ 0.8) were induced to differentiate akinetes by transferring them to low light condition (2–3 μmol photons m^–2^ s^–1^) with shaking at 50 rpm (Perez et al., 2016).

The germination of mature akinetes, exposed to different stress conditions, was induced by washing and transferring the culture to fresh BG11 media containing NaNO_3_ and optimal light conditions (Perez et al., 2018). The formation of akinetes and their germination was monitored with a Leica DM 2500 light microscope with an x100/1.3 oil objective, connected to a Leica DFC420C camera (Leica Microsystems GmbH, Wetzlar, Germany).

### Staining Procedures for Microscopy: Alcian Blue Staining

To observe the heterocyst envelope polysaccharide layer, Alcian blue staining was performed (Mckinney, 1953). Alcian blue solution [1.5% in H_2_O (w/v)] was added to the cell suspension (in a ratio of 1:100) and incubated at room temperature (RT) for 5–10 min.

### Triphenyl Tetrazolium Chloride (TTC) Staining

The culture containing heterocysts was mixed with the TTC solution [0.05% of TTC (w/v) in the final mixture] and incubated in dark for 15–30 min at RT (Fay and Kulasooriya, 1972).

### BODIPY Staining

In order to visualize the glycolipid layer in the heterocyst and akinete envelope, samples with heterocyst and akinetes were stained with boron-dipyrromethene difluoride (BODIPY) 493/503 (Molecular Probes, Thermo Fisher Scientific, Waltham, MA, United States) as described previously (Perez et al., 2016). After all staining procedures, filaments were placed on the slides covered with 1.5% agarose and observed by light microscopy with a Leica DM 2500 microscope connected to Leica DFC420C camera or with a Leica DM 5500B fluorescence microscope connected to Leica DFC420C camera. The green fluorescence signal was monitored with a BP470 40-nm excitation filter and a BP525 50-nm emission filter.

### Transmission Electron Microscopy (TEM)

For electron microscopy studies, individual cultures containing akinetes or heterocysts were fixed with 2.5% glutaraldehyde and post-fixed with 2% potassium permanganate followed by immobilization in agarose. After dehydration by successive increment of the ethanol concentration, the samples were embedded in Epon. Ultrathin sections were stained with uranyl acetate and lead citrate (Fiedler et al., 1998a), and examined using a Philips Tecnai 10 electron microscope at 80 kHz.

### RNA Isolation and Semi-Quantitative RT PCR (Reverse Transcription-PCR)

The total RNA was isolated at different time points after akinete induction (0, 3, 6, 12, and 18 days) or nitrogen step-down (0, 8, and 48 h) from wild-type and mutant DR752 cells, using UPzol reagent (Biotechrabbit, Henningsdorf, Germany) according to the manufacturer’s instructions. Briefly, cells were harvested at the different time points, immediately snap-freezed in liquid nitrogen and stored at −80°C till further use. Upon addition of 1 mL UPzol solution, cells were homogenized using glass beads and total RNA was extracted. Concentration and purity of the extracted RNA were estimated using nanophotometer (Implen) and RNA gel-electrophoresis followed by DNase treatment. The genomic DNA contamination was controlled by PCR followed by agarose gel electrophoresis. The DNase-treated RNA was used to generate cDNA using the RT-reaction kit (Applied Biosystems) and 1 μl of this cDNA was used for semi-q RT PCR followed by visualization on an agarose gel. Primers for semi-q RT PCR reactions are listed in Supplementary Table 2.

### Nitrogenase Activity

Nitrogenase activity was measured with the acetylene reduction method as previously described (Bornikoel et al., 2018; Shvarev et al., 2019). Briefly, after nitrogen step-down and 48 h cultivation under nitrogen-limiting conditions, the cell suspensions (20 μg Chl) were incubated under an atmosphere of 13.3% acetylene in air (oxic conditions) for several hours in flasks sealed with gas-tight caps and shaken in the light at 28°C. To generate anoxic conditions, a solution of 10 μM 3-(3,4-dichlorophenyl)-1, 1-dimethylurea (DCMU) (dissolved in methanol) was added and then, the sealed flasks were degassed, filled with argon and incubated for 1 h followed by incubation with acetylene. From each flask, 1 ml of the gaseous phase was taken, and the amount of ethylene produced was measured using gas chromatography.

### Analysis of Heterocyst and Akinete Specific Glycolipids

Thin-layer chromatography (TLC) was performed to analyze the glycolipids composition of heterocysts and akinetes as described (Winkenbach et al., 1972) with minor modifications. First, the chlorophyll *a* (Chl*a*) concentration was measured (Mackinney, 1941). Briefly, Chl*a* was extracted from 1 ml of cultures grown with (BG11) and without nitrogen source (BG11_0_), and from the cultures induced to form akinetes by low light for 2–4 months by adding methanol to a final concentration of 90% (v/v). The suspension was vortexed for 1 min and the cells were centrifuged at 13,000 rpm for 2 min at RT after incubation for 5 min in darkness followed by the measurement of adsorption of the supernatant at 665 nm. The final Chl*a* concentration was calculated using the formula:

Chl[μg/ml]=OD665×dilutionfactor× 13.43

For TLC, the cells were pelleted at equal Chl*a* concentration and resuspended in a methanol:chloroform mixture (1:1). Afterward, the lipids in the supernatant were concentrated by evaporation in air under a fume hood. The lipids were dissolved in chloroform and applied on an aluminum plate coated with silica gel (Macherey-Nagel, #818033). TLC was run in a mobile phase composed of chloroform:methanol:acetic acid:water in a ratio of 23:4:2.7:1. The lipids were visualized by spraying the TLC plate with 25% sulfuric acid and exposing it to 180°C for 60–120 s.

### Drop Assay for Resistance Test

The viability of akinetes (3–4 months old) was tested after exposing them to different stress conditions using an agar spot assay. For this, Chl*a* content of akinetes culture was determined as described in previous section. For inducing the akinetes to different stress conditions, 1 ml of akinete cultures with a concentration of 5 μg Chl*a* ml^–1^ were exposed to either freeze (−20°C) and cold (4°C) conditions or desiccated (centrifuged and pellet dried at 28°C) for 10, 20, and 30 days, respectively. Other stress conditions included the treatment with 10 mM H_2_O_2_ for 2 h at 28°C, with 300 μg ml^–1^ lysozyme overnight at RT in dark and the freeze (in liquid nitrogen for 5–7 min) and thaw (at RT for 20–30 min) cycles for three or six times. For each stress conditions and time points, various dilutions of akinete samples (in the range of 5 μg ml^–1^−0.5 μg ml^–1^ Chl*a*) were prepared and 10 μl of each dilution were carefully dropped on agar plates and incubated for 1 week at 28°C under continuous light.

## Results

### The *hglB* Gene Is Essential for Diazotrophic Growth of *Anabaena variabilis* ATCC 29413

By BLAST search, we identified the homologous *hglB* gene *Ava_2595* in the genome of *A. variabilis*. We created the mutant, which will be denoted DR752 from here onward, by insertion of a neomycin resistance cassette (CK.3) into the *Ava_2595* gene by double homologous recombination (Supplementary Figure 1A). The complete segregation of the mutated chromosome version in neomycin resistant clones was confirmed by colony PCR (Supplementary Figure 1B) and one correct clone was used for further studies.

**FIGURE 1:**
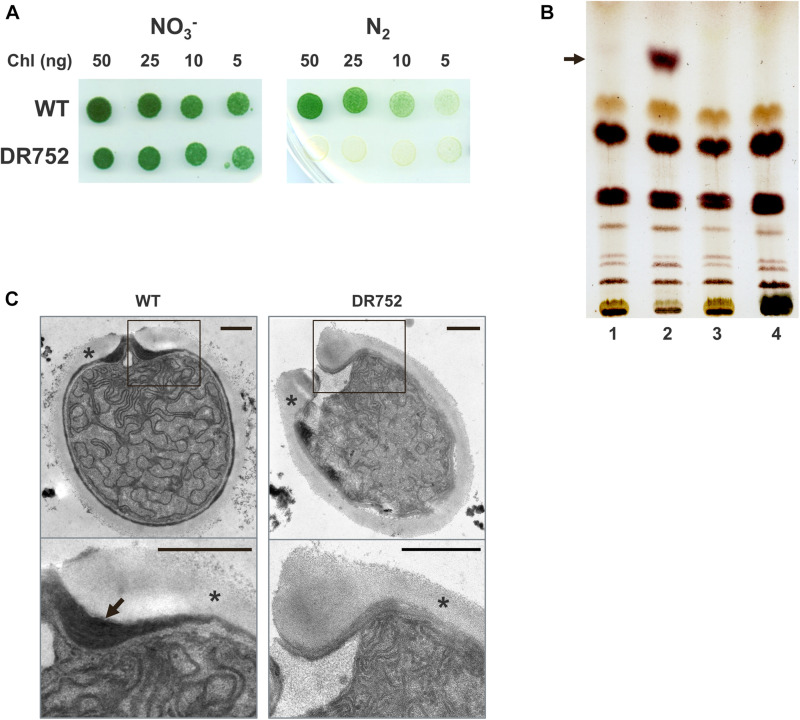
Growth of the mutant strain DR752 and analysis of heterocyst-specific glycolipids in the envelope. **(A)** The growth of *Anabaena variabilis* wild type (WT) and DR752 mutant colonies on solid AA/4 medium (NO3^–^) and medium lacking a combined nitrogen source (N_2_) after 7 days of incubation. **(B)** Thin-layer chromatography of lipid extracts from liquid cultures containing 50 μg of chlorophyll before and after nitrogen step-down for 72 h; 1-WT with nitrate, 2-WT without nitrate, 3-DR752 with nitrate, 4-DR752 without nitrate. The arrow indicates the position of one heterocyst-specific glycolipid. **(C)** Transmission electron micrographs of terminal heterocysts of WT and mutant DR752. The lower panels show the magnified view of the envelope, indicated by squares. Black arrow points toward the laminated layer and star indicates the exopolysaccharide layer. Bars, 0.5 μm.

Under standard growth conditions in media supplemented with NO_3_^–^, no significant differences in doubling time (Figure 1A), filament length and cell morphology were observed in DR752 mutant compared to the wild type (WT) (not shown). However, the mutant was not able to grow on NO_3_^–^-free medium (Figure 1A). In WT filaments, normally distributed heterocysts as well as terminal heterocysts were observed. In contrast, delayed heterocyst differentiation mostly by the terminal cells of the filaments was observed in mutant DR752 (Supplementary Figure 1C). After 2 days of nitrogen starvation, deposition of the outer polysaccharide layer in mutant heterocysts was confirmed by Alcian-blue staining, which stains the polysaccharide envelope in blue (Supplementary Figure 2A). In contrast, the envelope glycolipids could not be detected by staining with the fluorescent dye BODIPY (Supplementary Figure 2B), which stains the HGL layer of heterocysts and akinetes (Perez et al., 2016).

To investigate whether HGL is being synthesized in the mutant DR752 after nitrogen step-down, we performed thin-layer chromatography (TLC) with the lipids extracted from filaments of the WT and the DR752 strains. In contrast to the WT, the mutant did not show a band with the typical TLC migration property (Figure 1B), confirming that the *hglB* gene encodes a protein that is involved in HGL biosynthesis. This is in line with the semi-quantitative RT-PCR results showing that the *hglB* gene is differentially expresses in the WT strain during heterocyst differentiation (Supplementary Figure 3). As expected, a transcript of *hglB* could not be detected in the RNA of the mutant. Interestingly, the gene *hetN*, localized downstream of *hglB* is constitutively expressed in the mutant. In the WT, *hetN* was up regulated during nitrogen step-down (Supplementary Figure 3). Since the CK.3 cassette contains a strong promoter, but lacks a termination signal, we assume that the *hetN* gene, encoding a suppressor of heterocysts involved in pattern formation, gets under control of this constitutive promoter (Callahan and Buikema, 2001). Overexpression of *hetN* suppresses heterocyst differentiation in *Anabaena* sp. PCC 7120, which could explain the delayed heterocyst formation in the mutant DR752.

Furthermore, we studied the ultrastructure of the mutant heterocysts by transmission electron microscopy (TEM) of ultrathin sections of mutant and WT filaments (Figure 1C). As expected, the mutant did not form the laminated layer, explaining why it could not grow diazotrophically under oxic conditions.

**FIGURE 2:**
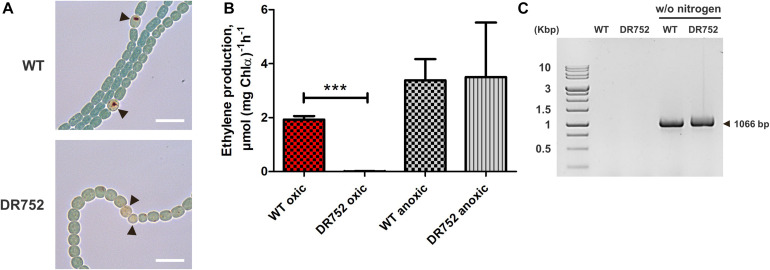
Nitrogenase activity and *nifHDK* operon rearrangement assessment. **(A)** Bright field images of nitrogen-starved filaments of WT and mutant DR752 after incubation with triphenyl tetrazolium chloride (TTC). Black arrowheads point to the heterocysts. TTC reduces to dark formazan crystals under microoxic conditions. Scale bar, 10 μm. **(B)** Assessment of the nitrogenase activity of WT and mutant DR752 under oxic and anoxic conditions. Filaments were incubated for 48 h in nitrogen-free medium before measuring the nitrogenase activity using the acetylene reduction assay. Data are representative of two independent experiments. The histogram shows the mean values ± standard deviation of three experimental replicates. Student’s *t*-test *P*-value is indicated as asterisks (***P < 0.0001). **(C)** The *nifD* rearrangement in WT and mutant DR752 after 72 h of nitrogen step-down was shown by PCR using primers (Ava903 FW and 919 Rv) left and right to the 11-kb insertion element as listed in Supplementary Table 2.

To assess the oxygen status in the heterocysts of the mutant, the filaments were incubated with triphenyl tetrazolium chloride (TTC), which forms dark red-brown precipitates in a microoxic environment (Fay and Kulasooriya, 1972). The WT heterocysts showed such precipitates but the DR752 mutant heterocysts did not (Figure 2A).

Furthermore, we used the acetylene reduction assay to measure the nitrogenase activity under oxic and anoxic conditions (Figure 2B). The DR752 mutant showed no nitrogenase activity under oxic conditions despite the occurrence of the *nifD* gene rearrangement required to produce a functional enzyme (Figure 2C; Brusca et al., 1989; Thiel and Pratte, 2014). In contrast, under anoxic conditions, the nitrogenase activity was measurable in the mutant and the WT at a similar level. This indicates that the mutant is not able to provide microoxic conditions suitable for the activity of the nitrogenase, as previously described for the *hglB* mutant of *Anabaena* PCC 7120 (Fox^–^, Fix^+^ phenotype).

### Mutant DR752 Is Affected in Akinete Differentiation

**FIGURE 3:**
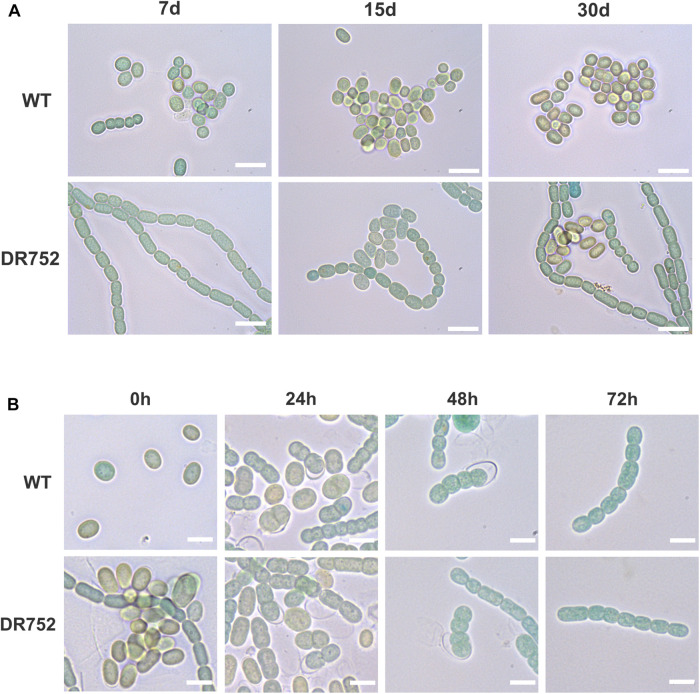
Akinete differentiation and germination process in WT and mutant DR752. **(A)** Bright field micrographs of the WT and mutant DR752 culture after 7, 15, and 30 days of akinetes differentiation in low light. Bars, 10 μm. **(B)** Germination of 3–4 months old mature akinetes was induced by transferring the cultures to normal light conditions and monitored by light microscopy after 0, 24, 48, and 72 h. Bars, 5 μm.

We have previously shown that the laminated layers of *A. variabilis* akinete mostly consist of the heterocyst-specific glycolipid HG_26_-diol (Perez et al., 2018). However, neither the function of this layer in akinete nor any enzyme for its synthesis was known so far. Hence, we investigated whether the *hglB* mutant from *A. variabilis* was affected in akinete differentiation and function. Stationary phase cultures of the WT and strain DR752 were incubated in low light conditions which trigger akinete formation (Perez et al., 2016). During the first 7 days in low light, WT filaments fragmented, whereas the mutant DR752 retained the long filaments and did not show any early sign of morphological differentiation (Figure 3A). After 15 days, approximately half of the WT filaments had turned into mature akinetes showing the typical morphology with the oval shape, thylakoid degradation evidenced by brownish color and a thick envelope (Perez et al., 2016). In contrast, the mutant strain showed only few immature akinetes with greenish color without a defined envelope (Figure 3A). After 30 days, most of the cells were mature akinetes in the WT culture. However, in the mutant DR752, approximately one-third of the mature akinetes were formed. This delayed akinete differentiation in DR752 indicates a possible role of HglB in akinete formation. However, upon a prolonged exposure of 2–4 months to low light, most of the vegetative cells in the DR752 mutant had finally differentiated into mature looking akinetes (not shown). Then, we investigated whether the akinetes of DR752 mutant were able to germinate similar to the WT akinetes. Three months after incubation of the filaments in low light, nearly all cells had differentiated to akinetes. After transferring these cultures to normal light, formation of filaments by germinating akinetes was observed. Cell division and resuscitation of the photosynthetic pigments occurred at the same speed in WT and mutant (Figure 3B).

### HglB Is Involved in the Formation of the Glycolipid Layer of the Akinete Envelope

**FIGURE 4:**
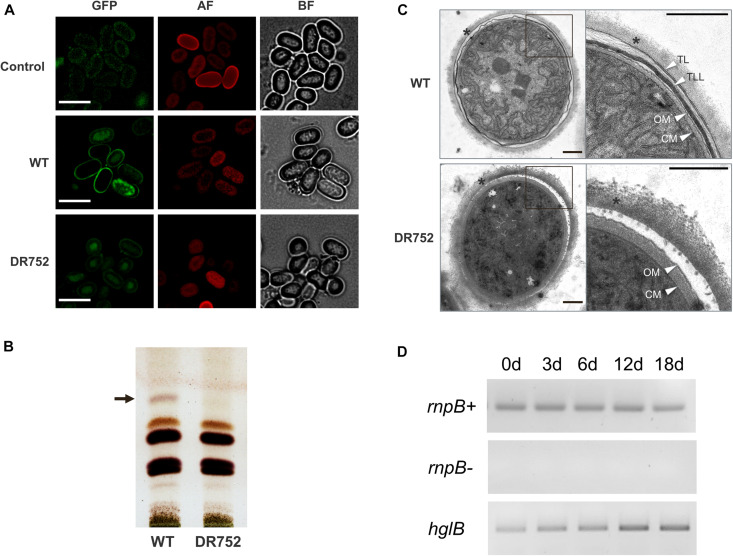
Analysis of glycolipids and expression of *hglB* during akinetes formation. **(A)** Akinete envelope stained with the green fluorescent dye BODIPY. Control, mutant akinetes without BODIPY staining; BF, bright field; GFP, green fluorescent protein filter; AF, autofluorescence red. Bars, 7.5 μm. **(B)** Thin-layer chromatogram of lipid extracts from liquid cultures induced to develop akinetes in low light for 4 months. The position of the glycolipid band corresponding to the heterocyst-specific glycolipid HG_26_-diol is indicated by an arrow. **(C)** Transmission electron micrographs (TEM) of akinetes of WT and mutant DR752. Right panels show the magnified view of akinete envelope, indicated by square. Star indicates the exopolysaccharide layer. White arrowheads indicate the following layers: TL, thin laminated layer; TLL, thick laminated layer; CM, cell membrane; OM, outer membrane. The empty space between outer membrane and polysaccharide layer is a dehydration artifact of the sample preparation procedure. Bars, 0.5 μm. **(D)** Expression analysis of *hglB* in the WT culture during akinete induction performed by semi-quantitative RT-PCR. *rnpB* (RNase P RNA coding gene) was used as a loading control with (*rnpB*+) and without reverse transcriptase (*rnpB*-). The primers used for semi-quantitative RT-PCR are RT rnpBFw, RT rnpBRv, RT hglBFw, and RT hglBRv; the sequences of the primers are listed in Supplementary Table 2.

Heterocysts and akinetes of several species have been suggested to possess a similar envelope structure and identical glycolipids (Nichols and Adams, 1982; Soriente et al., 1993; Leganés, 1994; Adams and Duggan, 1999; Perez et al., 2018). Two laminated layers are visible in electron micrographs of akinetes from *A. variabilis* (Figure 4C; see also Perez et al., 2016, 2018), and so far, it was not known, whether both consist of the same glycolipids. Therefore, we first compared the akinete envelopes of the WT with the mutant DR752 using the green fluorescent dye BODIPY, which stains the HGLs of akinetes and heterocysts. After cultivation in low light for 2–4 months, a strong well-defined green-fluorescent signal was detected in the outline of mature akinetes of the WT strain. In contrast, BODIPY did not stain the akinetes from the mutant, indicating the absence of lipid layers (Figure 4A).

To investigate the presence of glycolipids in the akinete envelopes of WT and mutant DR752, total lipids were extracted from cultures exposed to low light for 2–4 months and separated by TLC. In the WT extract, a lipid was detected at the position where HG_26_-ol from heterocyst migrates (Figure 4B), consistent with our previous study (Perez et al., 2018). This specific band was not observed in the DR752 mutant akinete culture, confirming that the *hglB* gene is involved in the synthesis of this glycolipid.

Next, we analyzed ultrathin sections of the akinetes of WT and DR752 mutant by TEM to solve the structure of the envelope at a better resolution. In the WT, two distinct electron dense laminated layers were visible between the outer exopolysaccharide layer and the outer membrane of the gram-negative cell wall [(Perez et al., 2016); Figure 4C]. Akinetes of the mutant DR752 showed the outermost polysaccharide layer, but both darkly stained laminated layers were absent. This confirms that the heterocyst lipid HG_26_-diol is also a component of these laminated layers, and that the HglB is essential for HGL-synthesis in akinetes. This is in line with the increased transcription of the *hglB*-gene during akinete differentiation as shown by semi-quantitative RT-PCR (Figure 4D).

### The Glycolipid Layer Is Important for Survival of Akinetes Under Various Stress Conditions

**FIGURE 5:**
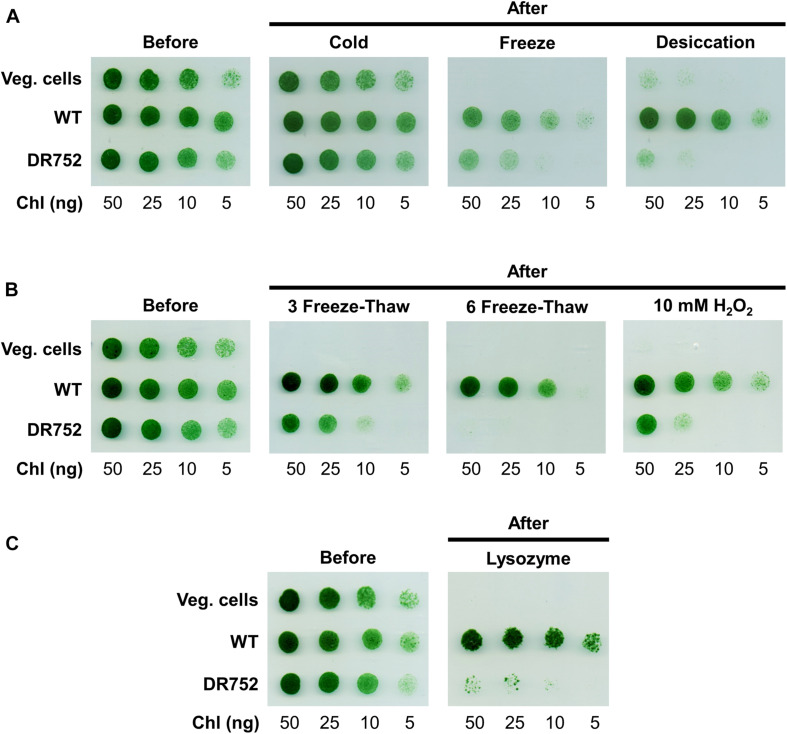
Viability of WT vegetative filaments and the akinetes of WT and mutant DR752 exposed to different stress conditions. Vegetative cells (Veg. cells) and 3–4 months akinete-induced culture of WT and mutant DR752 were exposed to/treated with **(A)** cold, freeze and desiccation for 20 days, **(B)** three and six freeze-thaw cycles or 10 mM H_2_O_2_ for 2 h and **(C)** 300 μg ml^–1^ lysozyme for overnight. Serial dilution and plating were performed on agar plates before and after treatments and incubated for 7 days to allow germination and pseudo colony formation.

Akinetes are highly resistant to various environmental stress factors such as cold, desiccation and nutrient starvation. However, little is known about the molecular basis for such resistance and the role of the special thick envelope (Kaplan-Levy et al., 2010). After having shown that the mutant DR752 has no laminated layers in their akinete envelopes, this mutant offered the opportunity to investigate the importance of the glycolipid layer in resistance and response to different environmental extremes. For this, the akinetes of WT and mutant DR752 along with the WT vegetative cells as control were exposed to various stress conditions for different time points before diluting and plating on non-selective agar plates to determine their survival by colony formation. The WT vegetative cells and the akinetes of WT and mutant DR752 showed similar survival and germination efficiency, respectively being treated (Figure 5, left panels). Surprisingly, the same survival efficiency was observed for all types of cells when exposed to low temperature (cold, 4°C) (Figure 5A). However, the vegetative filaments were highly susceptible to the other tested stress conditions. The DR752 mutant akinetes were significantly less resistant and suffered a decline in survival efficiency when subjected to freeze and desiccation, compared to WT akinetes (Figure 5A). Upon successive freeze-thaw cycles, the mutant DR752 akinetes showed a significant decrease in germination relative to the WT akinetes (Figure 5B). Interestingly, the mutant DR752 akinetes were particularly more susceptible and could not germinate upon exposure to six freeze-thaw cycles as compared to three cycles (Figure 5B). To determine the effect of oxidative stress, the WT and mutant DR752 akinetes were treated with 10 mM H_2_O_2_ for 2 h. In these conditions the mutant DR752 akinetes showed decreased resistance and survival compared to the WT akinetes (Figure 5B, right panel). Under these conditions, the mutant akinetes showed cell lysis. Similarly, when treated with 300 μg ml^–1^ lysozyme overnight, survival efficiency of the DR752 mutant akinetes was severely affected and only few colonies could germinate (Figure 5C). These results demonstrated that the mutant DR752 akinetes were more stable than the vegetative filaments but compared to the WT akinetes they were less resistant against various environmental stress conditions. Altogether, this study indicates that the glycolipid layer of the akinete envelope has an essential function in survival of akinetes.

## Discussion

Many genes are known to be involved in HGL formation in heterocysts of the model organism *Anabaena* sp. PCC 7120. In contrast, nothing is known about formation and role of the laminated layer in the akinete envelope. In this context, we have unraveled a new role of the homologous *hglB* gene in *A. variabilis* in glycolipid layer formation of the akinete envelope. As expected from the previously described phenotype of the *hglB*-mutant of *Anabaena* sp. PCC 7120, the DR752 mutant was also not able to grow on N_2_ as a sole nitrogen source and formed aberrant heterocysts lacking the laminated layer (Figures 1A,C). Since the DR752 mutant could not synthesize the HGLs (Figure 1B), we confirmed the previous assumption that HglB is an enzyme involved in HGL synthesis, even though the enzymatic activity of HglB has not been explored yet (Awai et al., 2009).

Consistent with the lack of the HGL layer, the mutant DR752 was not able to fix N_2_ in presence of oxygen, a phenotype described as Fox^–^ (Ernst et al., 1992; Campbell et al., 1997; Fan et al., 2006), because unable to provide microoxic conditions to protect the nitrogenase (Figure 2A). Consistently, nitrogenase activity was observed only under anoxic conditions. It has been shown that under anoxic conditions a second molybdenum-dependent nitrogenase is expressed also in vegetative cells of *A. variabilis* (Thiel et al., 1995, 2014), and is likely to contribute to a significant amount of activity under anoxic condition in the mutant as in the WT (Figure 2B). These observations support the non-functionality of the mutant heterocyst owing to the gas-permeability of cell envelope. The genome rearrangement within the *nifD*-gene (Brusca et al., 1989) is obviously not under control of oxygen, and can take place also in the mutant after withdrawal of a nitrogen source (Figure 2C).

Like heterocyst differentiation, akinete differentiation was delayed in the mutant and mature looking akinetes were detected much later compared to the WT (Figure 3A). Obviously, the state of the envelope formation has an influence on the maturation process of akinetes, but how this is sensed and influences the downstream signaling cascade needs to be further explored. However, an immediate downstream gene *hetN*, which was shown as a suppressor gene of heterocyst differentiation in *Anabaena* sp. PCC 7120 (Callahan and Buikema, 2001), was found to be constitutively expressed in DR752 mutant in contrast to the WT, where we observed its upregulation only after nitrogen step-down (Supplementary Figure 3). This may be due to the strong *psbA*- promoter of the CK.3 cassette (Elhai and Wolk, 1988) in the DR752 mutant, which might cause polar effects on the downstream genes. However, the *hetN* gene is not involved in glycolipid synthesis and we believe that its possible involvement can only be in delaying the heterocyst differentiation observed in the mutant. Whether *hetN* overexpression is also responsible for delayed akinete differentiation needs to be investigated in future.

For heterocyst envelope formation, it is well known that the envelope builds up in a highly regulated and ordered process [reviewed in: (Maldener et al., 2014; Flores et al., 2019)]. Electron micrographs and chemical characterization of the akinete envelope in *A. variabilis* and *N. punctiforme* showed that the laminated layer is composed of the heterocyst glycolipid HG_26_-diol (Perez et al., 2018). The HGLs are found in a plethora of heterocyst-forming cyanobacteria (Reddy, 1983; Gambacorta et al., 1998; Wörmer et al., 2012; Bauersachs et al., 2014), but they were also identified in akinetes of *Cyanospira rippkae* (Soriente et al., 1993), consistent with the perception that HGLs are not exclusive to heterocyst, but also found in akinetes (Wörmer et al., 2012). In addition, the outermost akinete envelope consists of the same polysaccharide material that comprises the HEP layer of the heterocyst envelope (Cardemil and Wolk, 1979; Leganés, 1994; Perez et al., 2018) thus, emphasizing the evolutionary and developmental relationships between these two specialized cells (Wolk et al., 1994).

In addition to the chemical and structural identity of the laminated layer of both cell types, our study indicates that akinetes and heterocysts use an overlapping biosynthetic pathway to produce the glycolipid envelope, involving the putative polyketide synthase HglB. To our knowledge, *hglB* (Ava_ 2595) from *A. variabilis* is the first known gene involved in the envelope formation of akinetes. In this respect, it would be interesting to analyze akinete formation of the previously described *hglE* mutant of *N. punctiforme*, which is lacking the HGLs in heterocysts and is affected in diazotrophic growth (Campbell et al., 1997).

To illustrate the importance of the glycolipid layers of the akinete cell envelope in stress tolerance and survival, we exposed the akinetes of WT and the *hglB*-mutant to unfavorable conditions. The significantly reduced resistance to freezing in the mutant, especially when using multiple freeze-thaw cycles, could be the consequence of a reduced cell wall stability (Figures 5A,B). Since, water expands when forming ice, the cell must withstand extreme pressure. In the mutant lacking the glycolipid layers the cell envelope is likely less stable. Indeed, the mutant akinetes and the vegetative cells lysed more easily upon freezing (visible by blue color of the medium; not shown), making them less viable in sub-zero environments. While the WT akinetes survived desiccation easily, the germination efficiency of the mutant was severely reduced (Figure 5A). This emphasizes the importance of the HGLs for resistance to desiccation, suggesting that the glycolipid layers prevent water efflux during dry periods. In bacteria, it is reported that desiccation causes hyperosmotic stress characterized by water loss, membrane disorganization and accumulation of reactive oxygen species and ultimately leads to the cell death (Potts, 1994; Greffe and Michiels, 2020). The reduced resistance to exogenous H_2_O_2_ could be explained by a thinner physical protective envelope in the mutant akinetes, which makes them more susceptible to damage by reactive oxygen species. The akinetes of the mutant are found to be less stable against any tested stress conditions, except the exposure to cold. Although the mutant develops defective akinetes, they are nevertheless more resistant to extreme environmental influences than vegetative cells due to the presence of the extracellular polysaccharide layer and intracellular adaptations (Argueta and Summers, 2005; Sukenik et al., 2015; Perez et al., 2016). Expectedly, the vegetative cells, which do not have an extra protecting cell envelope, were less resistant to any of the stress factors applied and only able to withstand cold for 20 days of incubation, conditions, which do not mimic cold winter seasons. In nature, cyanobacteria live in community with eukaryotic predators and other competing bacteria, which secrete proteases and cell wall lysing enzymes. Lysozyme, is a peptidoglycan degrading muramidase, which destroys the cell walls of bacteria (Ragland and Criss, 2017). The thick envelope of akinetes is capable to protect the dormant cells from lysozyme (Jensen and Sicko, 1971; Argueta and Summers, 2005) but the fully developed envelope with the glycolipid layers is a prerequisite for this (Figure 5C). Altogether, this study highlights the role of glycolipids in protecting the akinetes in harsh environments with changing conditions.

In summary, this study has established the diverse role of *hglB* in akinete and heterocyst envelope formation in *A. variabilis*. Additionally, we showed that glycolipid layers are necessary to protect the akinetes from various stress factors. The sharing of similar envelope-structures indicates that the developmental processes for akinetes and heterocysts occur in a similar way but fulfill different functions in these two specialized cell types of multicellular cyanobacteria. Further genes known to be involved in heterocyst envelope formation should be studied in the future to confirm the common biosynthetic pathways in heterocyst and akinetes and to learn more about their function in survival of dormant cells. It would also be interesting to understand the upstream divergent signaling pathways and to which point they converge.

## Data Availability Statement

The raw data supporting the conclusions of this article will be made available by the authors, without undue reservation.

## Author Contributions

RG designed and performed the experiments, interpreted the data, wrote most of the manuscript, drafted the work, made manuscript revisions, and gave final approval. IM designed and supervised the research, wrote part of the manuscript, made substantial contributions to the design of the work, and made interpretation of data for the work. Both authors contributed to the article and approved the submitted version.

## Conflict of Interest

The authors declare that the research was conducted in the absence of any commercial or financial relationships that could be construed as a potential conflict of interest.
