# Supplementary material for: The Dual Role of the Glycolipid Envelope in Different Cell Types of the Multicellular Cyanobacterium Anabaena variabilis ATCC 29413
Source: Front Microbiol. 2021 Apr 9;12:645028. doi: 10.3389/fmicb.2021.645028 (PMC8064123; doi:10.3389/fmicb.2021.645028)
Supplement: Supplementary file 1 [file Data_Sheet_1.pdf]

Supplemental Material

Table S1. Strains and plasmids used in this study.

Strain or plasmid	Source or reference
A. variabilis wild type	(Currier and Wolk, 1979; Thiel et al., 2014)
DR752	This study
pIM74	(Fiedler et al., 1998a)
pIM752	This study
Top10	Invitrogen
HB101 (pRL528)	(Wolk et al., 1984)
J53 (RP4)	(Wolk et al., 1984)
pRL271	(Black et al., 1993)

Table S2. Oligonucleotides used in this study in PCR.

All primers were purchased from Sigma-Aldrich.

Primer name	Sequence (5' to 3')
1979	GAAAGCTTGCATGCCTGCAATGAGTC TAAAACAAAATTATAG
1980	CATTGAGATCCTCTAGACGTACCAAA CAATATACATCAG
1981	GTATATTGTTTGGTACGTCTAGAGGA TCTCAATGAATATTG
1982	CATCAAATTGATAAAGTTCTAGAGGA TCCCCGGTGGGCGAAG
1983	CACCGGGGATCCTCTAGAACTTTATC AATTTGATGGTTAAG
1984	CAACGTTGTTGCCATTGCTCAAGCCA AGGTCAAGAACCCAG
Ava903 Fw	GTCCTCGTTTGGAAGGTAACAC
919 Rv	GAAGATACTGCGGAGCAAGGC
RT rnpBFw	GACCAGACTTGCTGGATAAC
RT rnpBRv	AAGACTCAAAATCCAAAATT
RT hglBFw	ACAGCAAACTCAGGCTGATG
RT hglBRv	CGCCAAAGTCTGGAACTGTG
RT hetNFw	ACAGGCAACGGTAGTTTGTG

RT hetNRv	AGGGCCGACAATATCTTGAG
2534	GGTAATGGGCTGTGGGCTTG
2535	CGCGTCTTCACCCGATTCAT

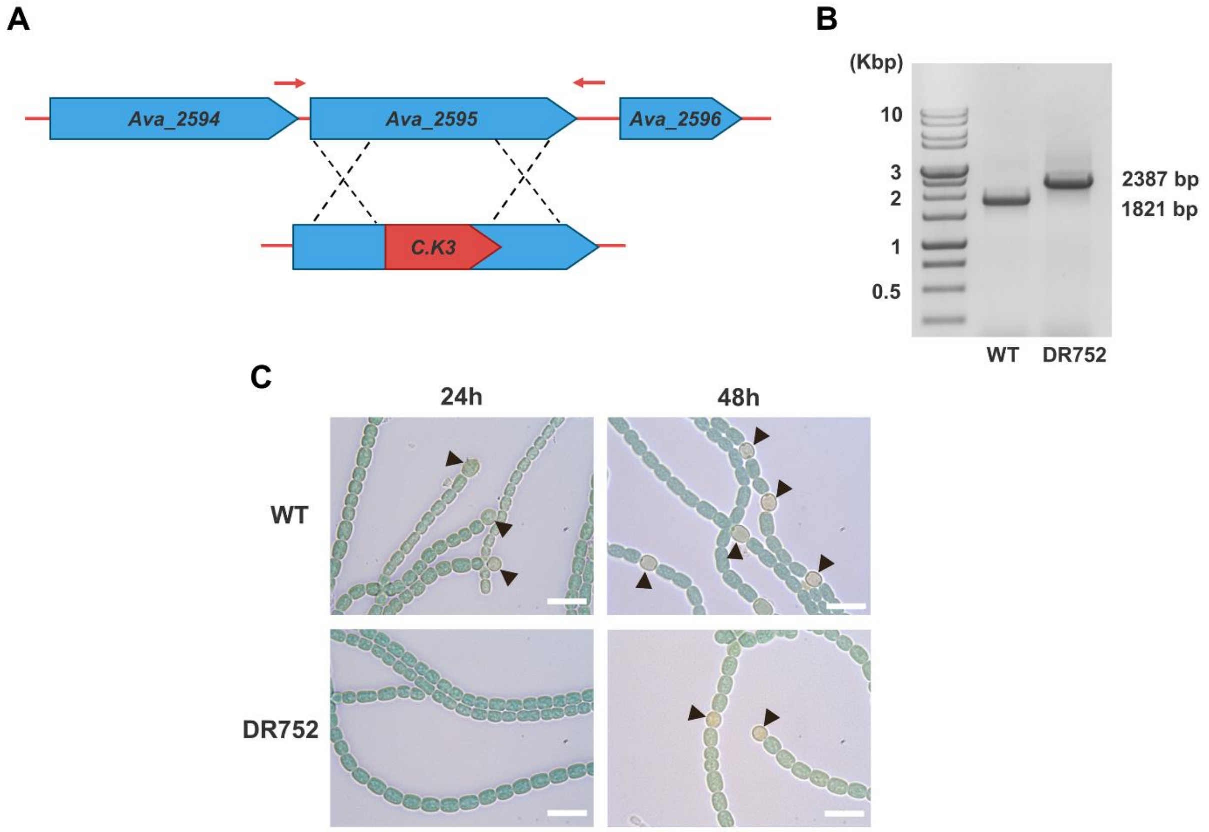

Figure S1 Construction and characterization of the *Anabaena variabilis* DR752 mutant. **(A)** Schematic of the strategy for the construction of mutant DR752. The *hglB* gene was disrupted by insertion of the neomycin resistance cassette (C.K3) into the genome by homologous recombination. Arrows indicate the location of primers used to verify complete segregation of the DR752 strain. **(B)** Confirmation of full segregation of the mutant by PCR. The amplification product of the wild type (WT) sequence is 1821 bp. After insertion, the sequence is 2387 bp. **(C)** Light micrographs of WT and mutant DR752 showing the heterocyst differentiation after 24 and 48 h of nitrogen step-down. Heterocysts are indicated by arrowheads. Bars, 10 µm.

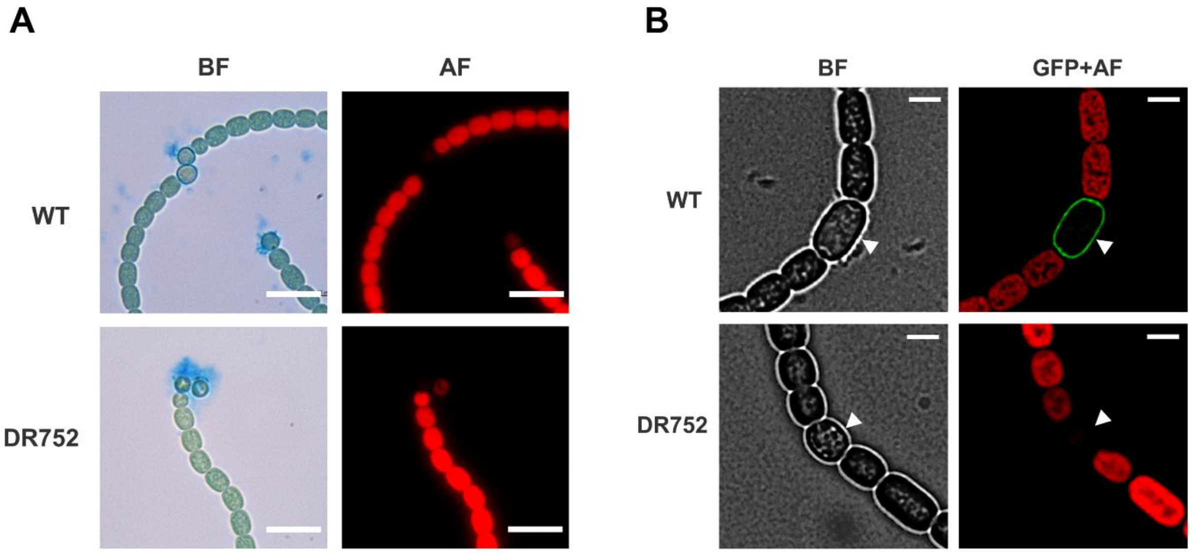

Figure S2 Alcian blue and BODIPY staining of the envelope of heterocyst**. (A)** Heterocysts envelope of WT and mutant DR752 stained with the Alcian blue dye (which stains exo-polysaccharides in blue) showed the presence of the polysaccharide layer. BF-bright field, AF-Autofluorescence. Bars, 10 µm. **(B)** Micrographs of filaments with heterocysts envelope stained with the green fluorescent dye BODIPY which binds to the neutral lipids. BF-bright field, GFP-green fluorescent protein filter, AF-Autofluorescence red. Heterocysts are indicated by white arrowheads. Bars, 3 µm.

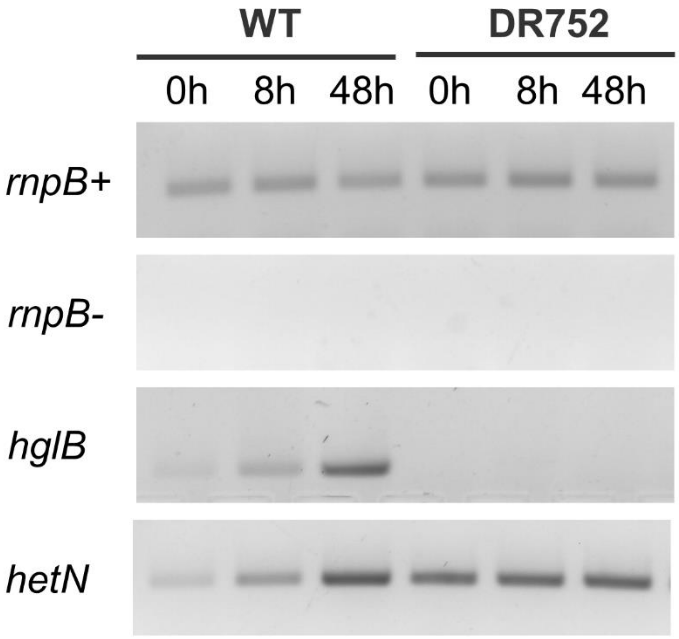

Figure S3 Analysis of gene expression during heterocyst differentiation. Time-dependent expression analysis of genes in the WT and mutant DR752 cultures during nitrogen starvation as assessed by semi-quantitative RT PCR. *rnpB* (ribonuclease B) was used as a loading control with (*rnpB*+) and without reverse transcriptase (*rnpB*-). Primers used for semi-quantitative RT PCR are listed in Table S2 in the supplemental material.
